# The neglected role of *Enterobius vermicularis* in appendicitis: A systematic review and meta-analysis

**DOI:** 10.1371/journal.pone.0232143

**Published:** 2020-04-23

**Authors:** Ali Taghipour, Meysam Olfatifar, Ehsan Javanmard, Mojtaba Norouzi, Hamed Mirjalali, Mohammad Reza Zali

**Affiliations:** 1 Department of Parasitology, Faculty of Medical Sciences, Tarbiat Modares University, Tehran, Iran; 2 Gastroenterology and Liver Diseases Research Center, Research Institute for Gastroenterology and Liver Diseases, Shahid Beheshti University of Medical Sciences, Tehran, Iran; 3 Department of Parasitology and Mycology, School of Medicine, Shahid Beheshti University of Medical sciences, Tehran, Iran; 4 Foodborne and Waterborne Diseases Research Center, Research Institute for Gastroenterology and Liver Diseases, Shahid Beheshti University of Medical Sciences, Tehran, Iran; Instituto Butantan, BRAZIL

## Abstract

Although the main cause of appendicitis is unclear, infection with *Enterobius vermicularis* is suggested as a neglected risk factor. Since, there is no comprehensive analysis to estimate the prevalence of *E*. *vermicularis* in appendicitis; therefore, we conducted a global-scale systematic review and meta-analysis study to estimate the prevalence of *E*. *vermicularis* infection in appendicitis cases. PubMed, Scopus, Web of Science and Google Scholar databases were systematically searched for relevant studies published until 15 August 2019. Pooled prevalence of *E*. *vermicularis* infection was estimated using the random effects model. Data were classified based on the continents and countries. Moreover, subgroup analyses regarding the gender, the human development index (HDI), and income level of countries were also performed. Fifty-nine studies involving 103195 appendix tissue samples belonging to the individuals of appendicitis were included. The pooled prevalence of *E*. *vermicularis* infection was (4%, 95%CI, 2–6%), with the highest prevalence (8%, 95% CI: 0–36%) and lowest prevalence (2%, 95% CI: 1–4%) in Africa and Americas continents, respectively. With respect to countries, the lowest and highest prevalence rates were reported from Venezuela (<1%, 95% CI: 0–1%) and Nigeria (33%, 95% CI: 17–52%), respectively. Indeed, a higher prevalence was observed in females, as well as in countries with lower levels of income and HDI. Our findings indicate the relatively high burden of *E*. *vermicularis* infection in appendicitis cases. However, our findings suggest the great need for more epidemiological studies to depth understand overlaps between *E*. *vermicularis* infection and appendicitis in countries with lower HDI and income levels.

## Introduction

Appendicitis is frequently reported from patients with severe abdominal pain requiring emergency surgery [1, 2]. According to the Global Burden of Disease (GBD) reports in 2015, approximately 11.6 million cases of appendicitis occurred with about 50100 deaths [3, 4]. The clinical manifestations of appendicitis commonly includes right lower abdominal pain, nausea, vomiting, and loss of appetite (anorexia) [5]. Despite recent progresses in antibiotic therapy, laparoscopic appendectomy has been remained a traditional treatment for acute appendicitis [6, 7].

There are various theories explaining the reasons of this disease; however, because of many factors contributed to appendicitis, the underlying cause is still unclear [1]. Interestingly, the role of infectious diseases in the etiology of acute appendicitis has remained controversial for more than one century [8, 9]. Some reports have spotlighted the probable relevance of appendicitis and infectious agents like *Fusobacterium* spp., [10] and herpes simplex virus [11]. Nevertheless, the nematode parasite, *Enterobius vermicularis*, has been proposed as a probable cause of appendicitis [12, 13].

*E*. *vermicularis* is a cosmopolitan parasite and one of the most common human-infecting helminths in temperate and cool climates, as well as developed countries [14, 15]. This parasite is usually transmitted through close-contact between infected and uninfected persons, ingestion and inhalation of the eggs [16]. Since, *E*. *vermicularis* has a simple transmission rout, re-infection is one of the main causes of development of the infection. However, complete life cycle of the helminth, from egg to adult worm, usually takes 2 to 4 weeks [17]. Although *E*. *vermicularis* infection commonly presents with perianal pruritus [18], it has also been reported to be associated with chronic abdominal pain, urinary tract infection, salpingitis, eosinophilic ileocolitis and pelvic abscess [19–22]. Couple of possible hypotheses explained the correlation between *E*. *vermicularis* and appendicitis of which mostly suggested ectopic migration of the parasite [23, 24]. Occasionally, erratic migration of eggs and larvae can elicit granuloma formation in the appendix [24], kidney [25], peritoneal cavity [26], male urinary tract [27], and female genital tract [28] which may lead to misdiagnosis. In the case of appendicitis, release and accumulation of eggs from female *E*. *vermicularis* may lead to the obstruction and inflammation of the appendix [29].

During recent decades, many articles have been published on the epidemiology and correlation of *E*. *vermicularis* and appendicitis, worldwide. In this global systematic review and meta-analysis, we assessed the status of *E*. *vermicularis* infection in appendicitis cases.

## Methods

### Information sources and search strategy

This review was done according to the preferred reporting items for systematic reviews and meta-analysis (PRISMA) [30]. International databases (Scopus, PubMed, Web of Science and Google Scholar) were searched for literature regarding the prevalence of *E*. *vermicularis* in individuals with appendicitis (from their inception until August 15, 2019), relevant papers were found using the following search terms: (“*Enterobius vermicularis*” OR “*E*. *vermicularis*” OR “Enterobiasis” OR “Oxyure” OR “*Oxyuris vermicularis*” OR “Oxyuriasis” OR “Pinworm” OR “Roundworm” OR “Threadworm” OR “Seatworm”) AND (“Appendix” OR “Appendices” OR “Appendicitis” OR “Appendectomy”) AND/OR (“Prevalence” OR “Frequency”). The bibliographic list of the relevant studies and reviews were explored in depth to find other related literatures which were not found via database searching.

### Eligibility criteria, study selection and data extraction

Literature was initially screened by title and abstract, and after duplicate removal, the full text of eligible entries was retrieved via online resources. Two trained investigators evaluated the eligibility (AT and MN), then any discrepancies were obviated by consensus and discussion with a third reviewer (HM). The final required data were extracted by two authors and rechecked by third author (HM), as follows: the first author, implementation and publication year, country, continent, gender, diagnostic method, study design, total sample size, and number of infected subjects in studies. In addition, we collected information on HDI (http://hdr.undp.org/en/composite/HDI) and income level (https://datahelpdesk.worldbank.org/knowledgebase/articles/906519-world-bank-country-and-lendinggroups) of each country.

Inclusion criteria for our systematic review and meta-analysis were: (1) Peer-reviewed original articles and short reports, without geographical and time limitations; (2) studies published with full text or abstracts in English; (3) Studies conducted until August 15, 2019; (4) having total sample size and positive samples in appendicitis cases (5); we selected the confirmed cases of *E*. *vermicularis* infection by histopathological methods, such as presence of eggs or larvae worms in appendix. Articles without any of aforementioned criteria including reviews, editorials and/or letters, those with confusing/unclear analyses, and those with a specific population (*e*.*g*. the general population and immunocompromised groups) were dismissed.

### Data synthesis and statistical analysis

In the present study, all statistical analyses were conducted using Meta for packages of R software version 3.5.1. The prevalence of *E*. *vermicularis* infection in appendicitis cases at a 95% confidence interval (CI) was estimated using a random effect model. Heterogeneity between studies was assessed using I^2^ methods. I^2^ values of 25%, 50% and 75% were considered as low, moderate and high heterogeneity, respectively. The pooled estimates were stratified based on the continents and countries. Moreover, subgroup analyses were conducted according to gender, income level and HDI of countries. In order to investigate the possibility of publication bias during the analysis, Eggers regression was employed. A *P*-value of less than 0.05 was considered statistically significant.

## Results

**Fig 1:**
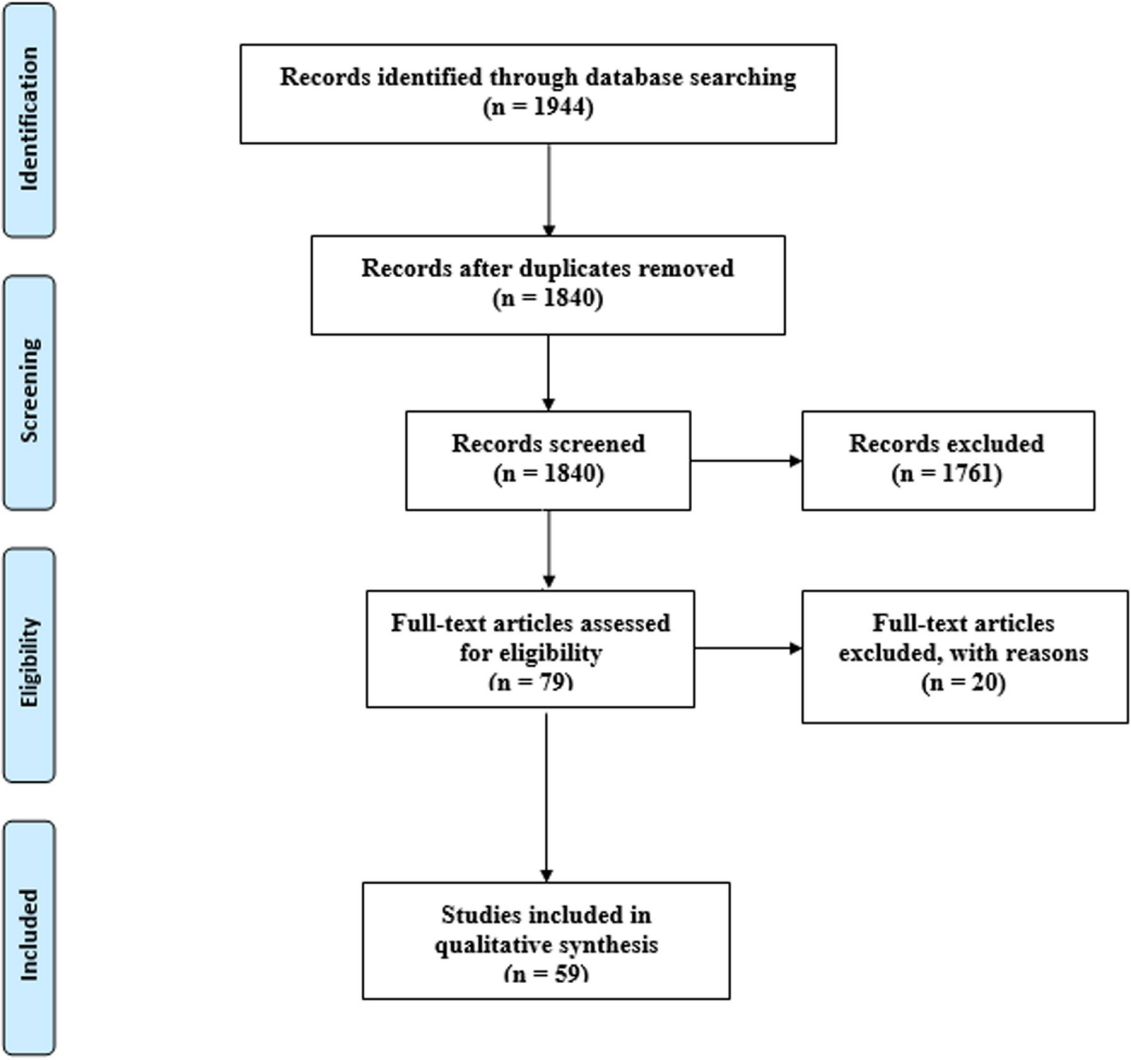
Flow diagram of the study design process.

As shown in Fig 1, a total of 1944 papers were found following the initial search of databases and ultimately 59 articles from 24 countries out of five continents met the inclusion criteria in the systematic review and meta-analysis [31–89] (Fig 1, S1 Table). Totally, 103195 appendix tissue samples belonging to the appendicitis cases were evaluated for *E*. *vermicularis* infection from Dec 1939 to Aug 2019 of which 2983 (2.89%) patients were positive for the helminth. The main study characteristics, sample size, and positive rate of *E*. *vermicularis* infection in appendicitis cases are presented in Table 1.

**Table 1:** Main characteristics of all eligible studies reporting prevalence *E*. *vermicularis* in appendicitis.

First author/ Continent/ Ref	Publication year	Country	HDI	Income level	Total Sample	Infected Sample
**Europe**						
**Duran-Jorda**	1956	UK	Very high	High	691	52
**Boulos and Cowie**	1973	UK	Very high	High	293	8
**Sterba and Vlcek**	1984	Czech Republic	Very high	High	21916	1321
**Budd and Armstrong**	1987	UK	Very high	High	1529	38
**Bredesen et al.**	1988	Denmark	Very high	High	303	38
**Wiebe**	1991	Denmark	Very high	High	2267	93
**Listorto et al.**	1996	Italy	Very high	High	1093	14
**Saxena et al.**	2001	Germany	Very high	High	62	3
**Yildirim et al.**	2005	Turkey	High	Upper middle	104	4
**Isik et al.**	2007	Turkey	High	Upper middle	890	18
**Aydin et al.**	2007	Turkey	High	Upper middle	190	4
**Sodergren et al.**	2009	UK	Very high	High	1150	18
**Karatepe et al.**	2009	Turkey	High	Upper middle	5100	12
**Ariyarathenam et al.**	2010	UK	Very high	High	498	13
**Engin et al.**	2010	Turkey	High	Upper middle	1969	7
**Karaman et al.**	2010	Turkey	High	Upper middle	916	23
**Akbulut et al.**	2011	Turkey	High	Upper middle	54	37
**Mekhail et al.**	2011	UK	Very high	High	268	8
**Gialamas et al.**	2012	Greece	Very high	High	1085	7
**Yilmaz et al.**	2013	Turkey	High	Upper middle	134	31
**Ilhan et al.**	2013	Turkey	High	Upper middle	3863	16
**Yabanoglu et al.**	2014	Turkey	High	Upper middle	57	15
**Yabanoglu et al.**	2014	Turkey	High	Upper middle	1159	15
**Fleming et al.**	2015	Ireland	Very high	High	182	13
**Yıldiz et al.**	2015	Turkey	High	Upper middle	846	12
**Akkapulu and Abdullazade**	2016	Turkey	High	Upper middle	1446	9
**Gorter et al.**	2017	Netherlands	Very high	High	484	5
**Altun et al.**	2017	Turkey	High	Upper middle	660	9
**Dincel et al.**	2018	Turkey	High	Upper middle	1970	11
**Unver et al.**	2019	Turkey	High	Upper middle	2047	4
**Tayfur and Balci**	2019	Turkey	High	Upper middle	2400	22
**Pehlivanoglu et al.**	2019	Turkey	High	Upper middle	3222	24
**Oztas et al.**	2019	Turkey	High	Upper middle	48	37
**Americas**						
**Botsford and Hudson**	1939	USA	Very high	High	1343	71
**Ashburn**	1941	USA	Very high	High	1319	133
**Wax and Cooper**	1941	USA	Very high	High	1016	8
**Dorfman et al.**	1995	Venezuela	High	Upper middle	3465	14
**Agarwala and Liu**	2003	USA	Very high	High	317	1
**Arca et al.**	2004	USA	Very high	High	1549	21
**Di et al.**	2006	Argentina	Very high	High	186	2
**da Silva et al.**	2007	Brazil	High	Upper middle	1600	23
**Maki et al.**	2012	USA	Very high	High	913	16
**Alemayehu et al.**	2014	USA	Very high	High	3602	34
**Spitale et al.**	2017	Argentina	Very high	High	2000	65
**Asia**						
**Babekir and Devi**	1990	United Arab Emirates	Very high	High	405	26
**Dalimi and Khoshzaban**	1993	Iran	High	Upper middle	1590	38
**Fallah et al.**	2006	Iran	High	Upper middle	5981	38
**Sah and Bhadani**	2006	Nepal	Medium	Low	624	9
**Ramezani and Dehghani**	2007	Iran	High	Upper middle	5048	144
**Zakaria et al.**	2013	Saudi Arabia	Very high	High	1600	45
**Ahmed et al.**	2015	Pakistan	Medium	Lower middle	2956	84
**Zaghlool et al.**	2015	Saudi Arabia	Very high	High	1536	4
**Hamdona et al.**	2016	Palestine	Medium	Lower middle	200	30
**Africa**						
**Okolie et al.**	2008	Nigeria	Low	Lower middle	27	9
**Limaiem et al.**	2015	Tunisia	High	Lower middle	1627	23
**Zouari et al.**	2018	Tunisia	High	Lower middle	540	53
**Amer et al.**	2018	Egypt	Medium	Lower middle	65	1
**Oceania**						
**Dahlstrom and Macarthur**	1994	Australia	Very high	High	1867	63
**Lala and Upadhyay**	2016	New Zealand	Very high	High	2923	109

**Abbreviations:** HDI: human development index.

Regarding the income level, 28, 24, six, and one studies were conducted in countries with high, upper middle, lower middle, and low levels of income, respectively. Considering the HDI, 28, 26, four, and one studies were performed in countries with very high, high, medium, and low levels of HDI. Thirteen studies had extractable data regarding the gender (including 8201 males and 8375 females). The random-effects model was used due to the presence of significant heterogeneity (I^2^ = 98%). Detecting publication bias using the Eggers regression revealed that publication bias was statistically very significant (*P*< 0.000).

**Fig 2:**
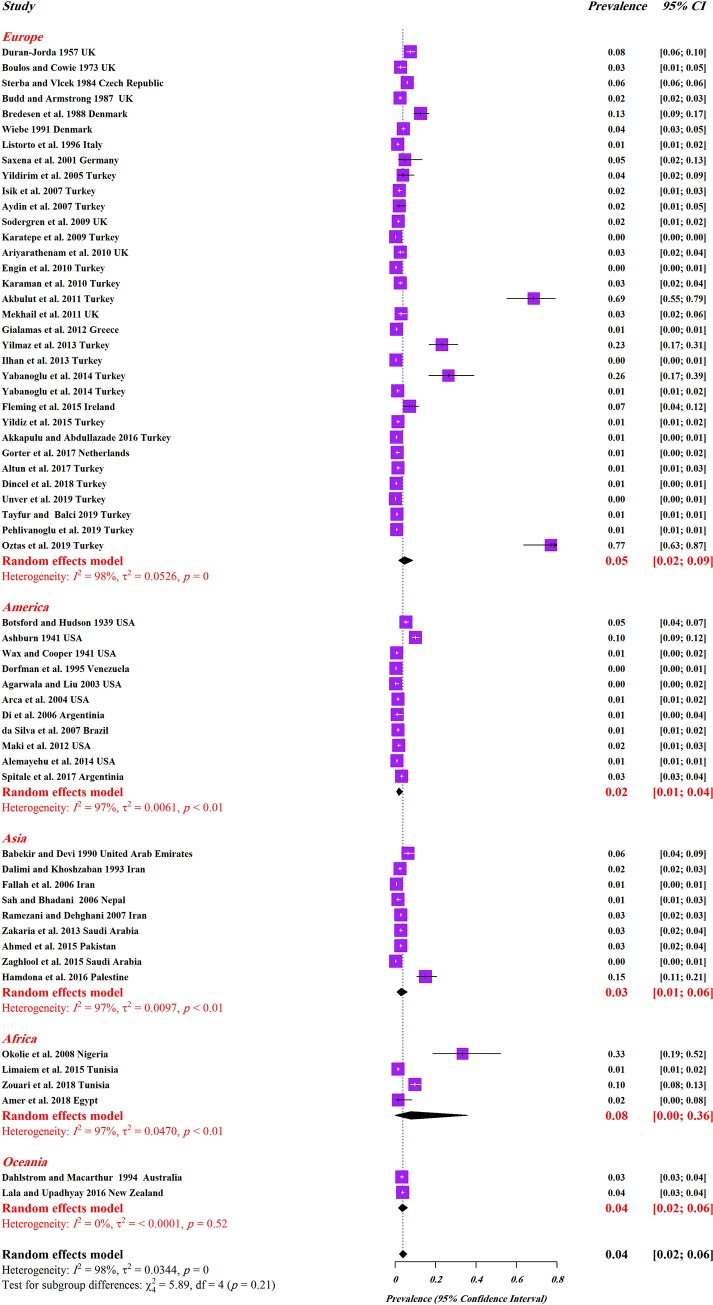
Forest plots for random-effects meta-analysis of *E*. *vermicularis* in appendicitis based on the prevalence in different continents.

The overall prevalence of a positive histopathological methods result for *E*. *vermicularis* infection in appendicitis cases was estimated to be (4%; 95%CI, 2–6%) (Fig 2 and Table 2). The highest and lowest global burdens of *E*. *vermicularis* infection were found in the continents Africa (8%; 95%CI, 0–36%) and Americas (2%, 95%CI: 1–4%), respectively (Fig 2 and Table 2). Nigeria (33%, 95% CI: 17–52%) was identified as a country with the highest percentage of histopathological positive results while the lowest prevalence (<1%, 95% CI: 0–1%) was found in Venezuela (S1 Fig).

**Table 2:** Sub-group analysis of the prevalence of *E*. *vermicularis* based on continents, HDI and income level, and gender.

Variable/sub-groups	Number of studies	Sample size	Infected	Pooled prevalence (95% CI)	Heterogeneity	
					*P* value	I^2^
**Continent**						
Europe	33	58896	1941	2.7 (1.8–4)	0.00	97.5
Americas	11	17310	388	1.6 (0.8–3.1)	0.00	97.2
Asia	9	19940	418	2.4 (1.4–4.1)	0.00	96.2
Africa	4	2259	86	6 (1.4–22.8)	0.00	96.7
Oceania	2	4790	172	3.6 (3.1–4.2)	0.52	0.00
Overall	59	103195	3005	3.3 (2.9–3.8)	0.00	97.2
**HDI**						
Very high	28	52397	2229	2.7 (2.1–3.5)	0.00	95.2
High	26	46926	643	2.1 (1.2–3.8)	0.00	97.8
Medium	4	3845	124	3.5 (1.1–10.9)	0.00	95.9
Low	1	27	9	33.3 (18.3–52.7)	1	0.00
**Income level**						
High	28	52397	2229	2.7 (2.1–3.5)	0.00	95.2
Upper middle	24	44759	567	2 (1.1–3.8)	0.00	97.8
Lower middle	6	5415	200	6.4 (2.6–14.7)	0.00	96.8
Low	1	624	9	1.4 (0.8–2.7)	1	0.00
**Gender**						
Male	11	8201	164	2.7 (2.3–3.2)	0.00	93.8
Female	13	8375	320	4.9 (2.9–8.1)	0.00	94.2

**Fig 3:**
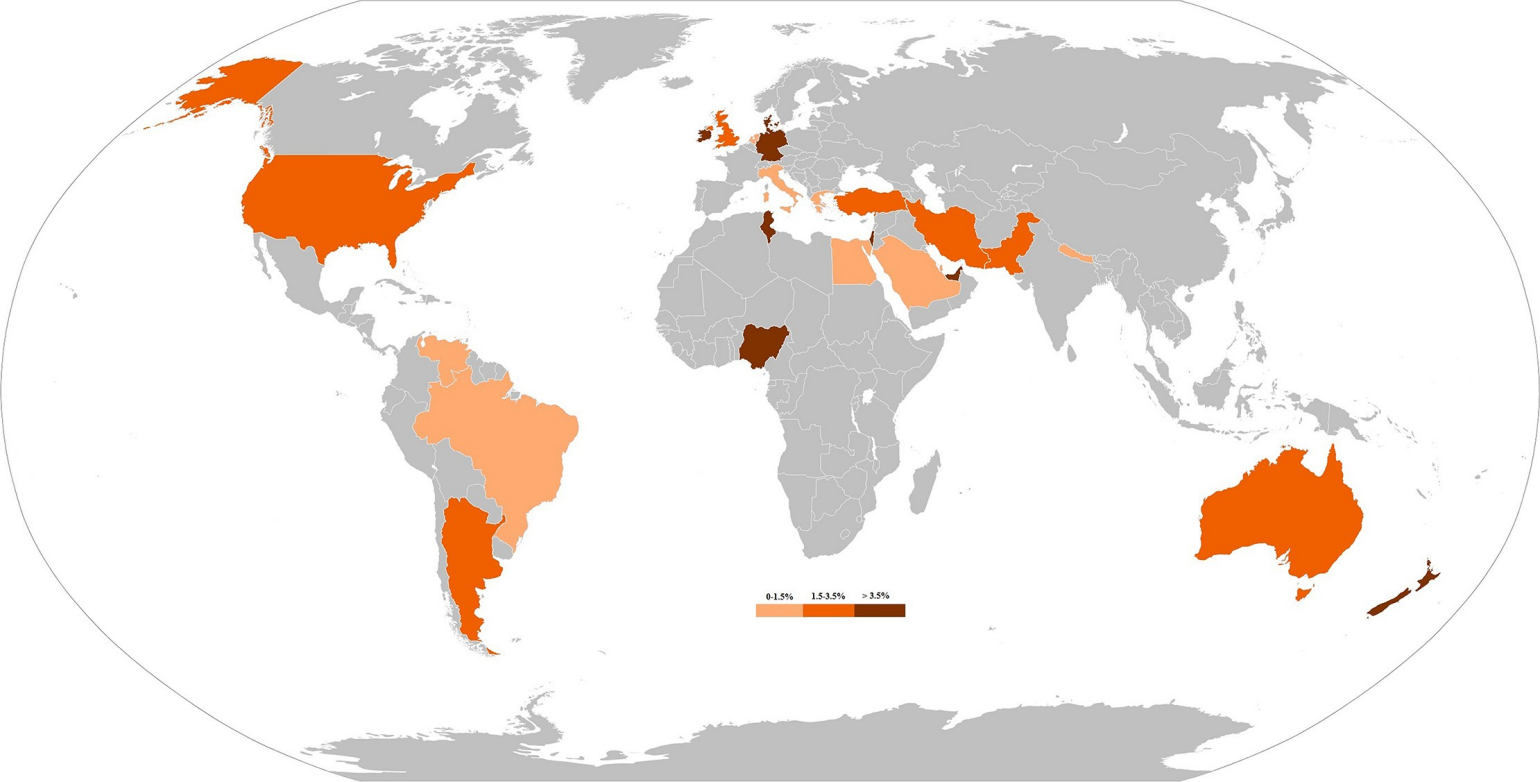
The prevalence of *E*. *vermicularis* appendicitis cases from different countries. This map shows that the prevalence rate of the parasite is mostly ranged < 3.5%. All figures were produced by the authors specifically for this manuscript. The raw map was downloaded from a free web source: https://commons.wikimedia.org/wiki/Atlas_of_the_world and edited with Photoshop cc by Ehsan Javanmard and Hamed Mirjalali.

The prevalence map of *E*. *vermicularis* infections in appendicitis cases from different countries is presented in Fig 3. In a subgroup analysis by income level, the estimated prevalence of *E*. *vermicularis* infection in countries with high, upper middle, lower middle, and low levels of income was (3%, 95%CI: 2–4), (4%, 95%CI: 1–10%), (8%, 95%CI: 1–21%) and (1%, 95%CI: 1–3), respectively (Table 2, Fig 3 and S2 Fig). With regard to HDI, meta-analysis results revealed that the prevalence of *E*. *vermicularis* infection in countries with very high, high, medium, and low HDI was (3%, 95%CI: 2–4), (4%, 95% CI: 1–10%), (4%, 95% CI: 0–16%) and (33%, 95%CI: 17–52), respectively (Table 2 and S3 Fig).

**Fig 4:**
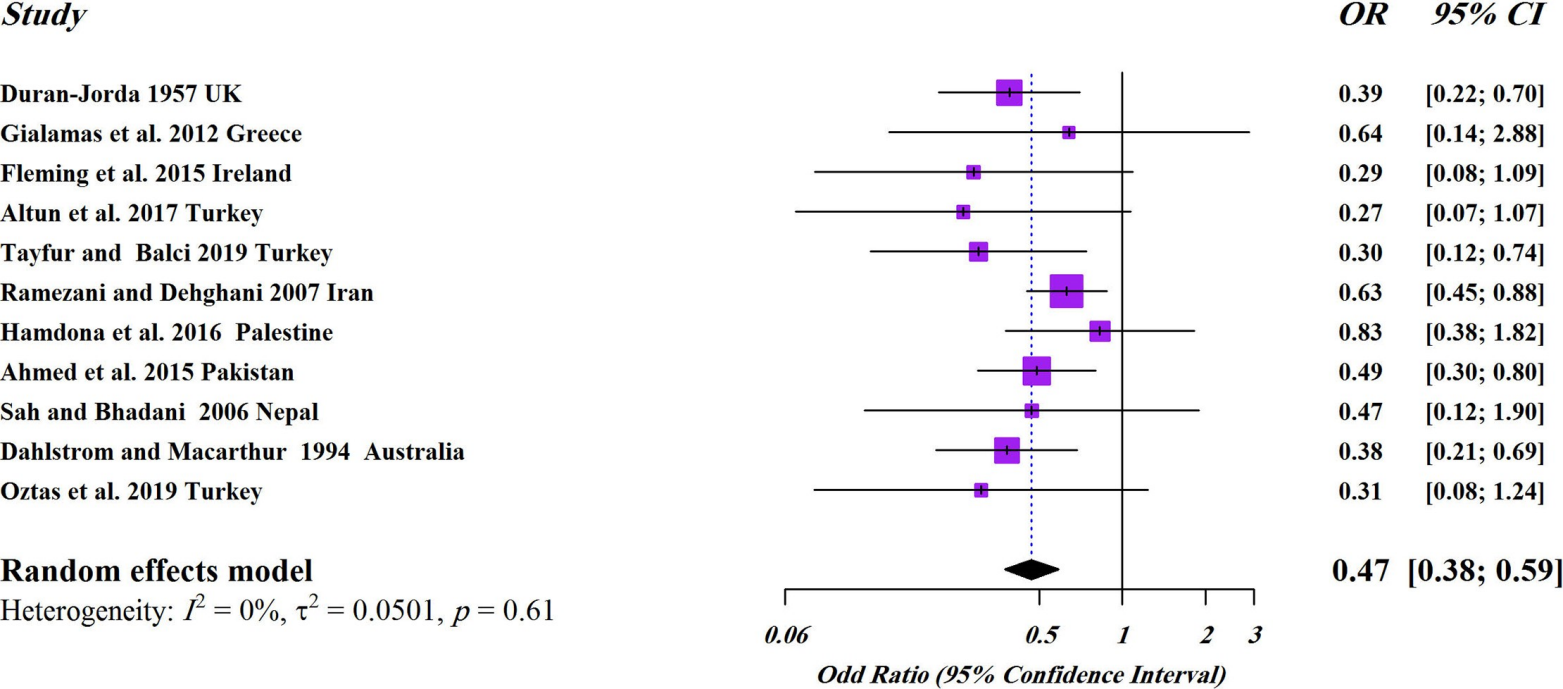
Forest plot pooled with random effects regarding the prevalence of *E*. *vermicularis* in appendicitis cases showing the OR and 95% CI by sub-group based on gender.

In a subgroup analysis by gender, the pooled prevalence in females 4.9% (2.9–8.1) was higher than males 2.7% (2.3–3.2) (Table 2), showing a statistically significant difference (OR, 0.47; 95%CI, 0.38–0.59) (Fig 4).

## Discussion

Considering the fact that *E*. *vermicularis* infection is one of the important neglected causes of inflammation of appendix, it is necessary to discuss our knowledge about the prevalence rate of this infection in appendicitis cases. The presence of *E*. *vermicularis* infection in appendicitis was firstly reported by Fabrius in 1634 [90]. Since then, researchers have performed many studies on this line [36, 38, 91]. This systematic review is the first of that brings information to reveal global status of *E*. *vermicularis* infection in appendicitis cases. Our findings could be helpful for physicians and public health policy makers, especially in countries with lower health levels.

Our results indicated that 3005 tissue samples out of 103195 appendicitis cases were positive for *E*. *vermicularis* infection. We observed a geographical variation for the prevalence of *E*. *vermicularis* infection in appendicitis cases ranging from approximately 2% in the Americas to 8% in Africa. This variation in different continents could be resulted from lifestyle, sanitation status, culture, socioeconomic conditions, and climate [92–95]. For this purpose, we have done two sub-group analyses to evaluate the impact of HDI and income level parameters on the prevalence of *E*. *vermicularis*. As a result, low-income countries with lower HDI had higher prevalence of *E*. *vermicularis* than high-income countries with higher HDI.

In many territories, the prevalence of *E*. *vermicularis* has significantly decreased in recent decades due to screening programs and improved public health levels. For example, this reduction was observed in Turkey (from 45.9% to 16%) [96], Greece (from 22.1% to 5.2%) [97] and South Korea (from 17.1% to 7.9%) [98, 99]. However, although the global prevalence of helminthic infections reduced during the recent decades, it seems that regarding this fact that enterobiasis is a benign infection and most of infected subjects are asymptomatic, most of cases might be misdiagnosed during the screening programs.

The sub-group analysis showed that the prevalence of *E*. *vermicularis* in females was significantly higher than males (OR, 0.47; 95%CI, 0.38–0.59). Higher infection rates among females could be attributed to different behavioral patterns, as well as gender-based differences. Actually, housewife females usually work in kitchen and have close-contact to raw vegetables that makes them more prone to be infected with parasite (oo)cysts and eggs. On the other hand, it is interesting to mention that *E*. *vermicularis* was commonly seen in girls with average age of 12 years [36, 69] that makes them more susceptible to ectopic infections such as vulvitis and vaginitis.

Based on different aspects of histopathological variations, most of studies have shown a relatively high frequency of infiltration of neutrophils and purulent exudate as the most commonly observed findings [81, 100]. Moreover, eosinophilia, fecaliths, and the eggs in the lumen might be the microscopic reasons for appendicitis due to *E*. *vermicularis* [81, 101]. However, some studies concluded that mucosal infiltration by the eggs was not a factor for appendicitis [88]. Therefore, it should be considered that the role of *E*. *vermicularis* infection in appendicitis is still controversial [102]. Nevertheless, in appendicitis cases that no causative (probable) agents were detected except *E*. *vermicularis*, the neglected role of this helminth should be considered.

The most important strengths of this systematic review and meta-analysis study are performing a comprehensive search of articles in four international databases, robust methodology, and conducting several subgroup analyses. Furthermore, this study has some limitations and the results presented here should be interpreted with regard to them including: 1) low number of researches in the case of the prevalence of *E*. *vermicularis* in appendicitis cases for many parts of the world and high heterogeneity. Moreover, in majority of the included articles, risk and demographic factors were not evaluated.

To minimize these limitations, we recommend that a standard questionnaire should be designed in order to perform a more comprehensive judgment on the risk factors including: gender, age, residence, education level, and occupation. Finally, we suggest that researchers should focus on the understanding the overlap between the presence of *E*. *vermicularis* and appendicitis in parts of the world, where there is a lack of information on the epidemiological aspects of *E*. *vermicularis* in appendicitis cases.

## Conclusion

In conclusion, the results of the current study indicated that *E*. *vermicularis* is one of the common infectious agents that could be found in the appendix and may increase the risk of appendicitis. In addition, we concluded that HDI and socioeconomic conditions probably have direct effects on the prevalence of *E*. *vermicularis*, as well as appendicitis. This finding highlights the importance for considering the neglected role of parasites in some clinical cases such as appendicitis. Consequently, the possibility of intestinal parasitic infection of the appendix should be considered in the differential diagnosis of agents that may be involved in appendicitis. Moreover, it seems that stool and scotch adhesive tape examination for intestinal parasites should be incorporated into the routine screening of appendicitis, especially for helminths.

## Supporting information

S1 Fig Forest plots for random-effects meta-analysis of *E*. *vermicularis* in appendicitis based on the prevalence of the infection in different countries. (JPG)

S2 Fig Forest plots for random-effects meta-analysis of *E*. *vermicularis* in appendicitis based on the prevalence of the infection in different income levels. (JPG)

S3 Fig Forest plots for random-effects meta-analysis of *E*. *vermicularis* in appendicitis based on the prevalence of the infection in different HDI. (JPG)

S1 Table Prisma checklist. (DOC)

## Abbreviations

HDI: human development index
PRISMA: preferred reporting items for systematic reviews and meta-analysis
CI: confidence interval
OR: odds ratio
