# Supplementary figures and images for: The neglected role of Enterobius vermicularis in appendicitis: A systematic review and meta-analysis
Source: PLoS One. 2020 Apr 23;15(4):e0232143. doi: 10.1371/journal.pone.0232143 (PMC7179856; doi:10.1371/journal.pone.0232143)

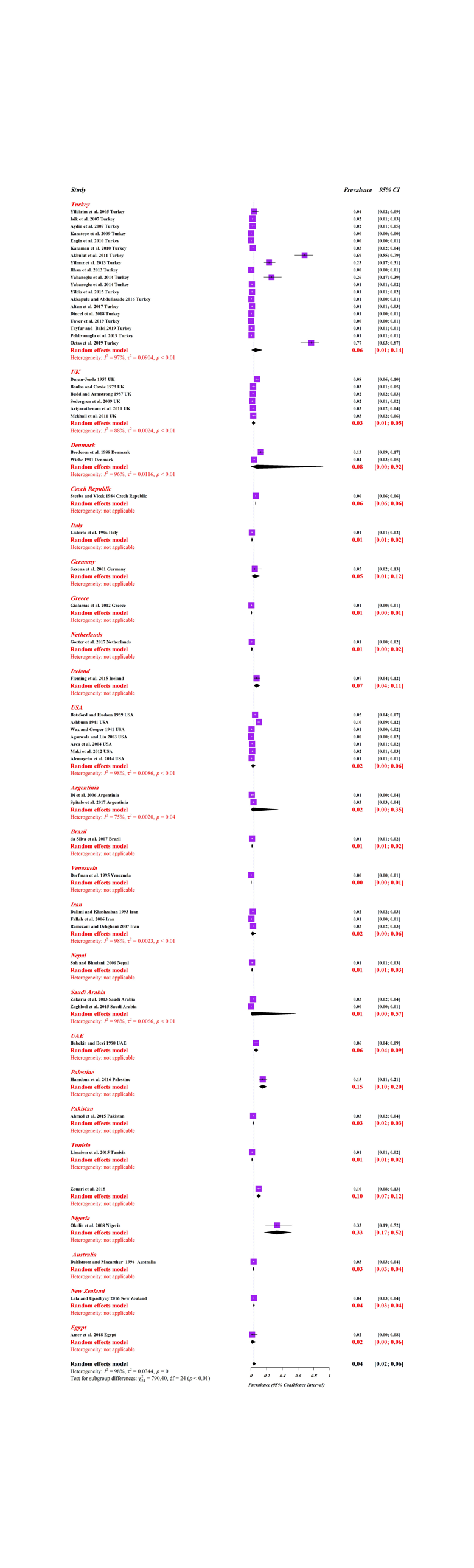

Supplement: S1 Fig — (JPG) [file pone.0232143.s001.jpg]

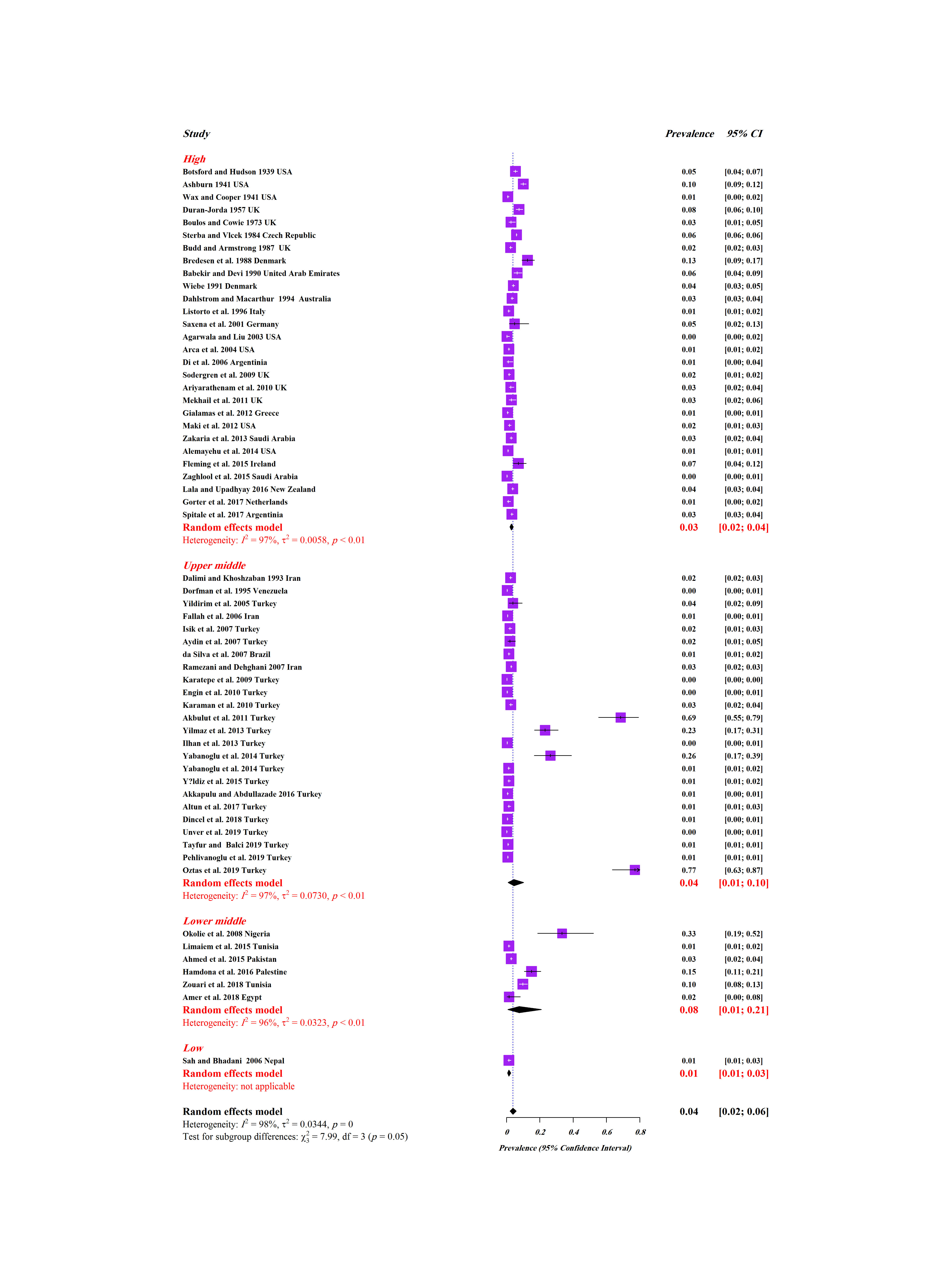

Supplement: S2 Fig — (JPG) [file pone.0232143.s002.jpg]

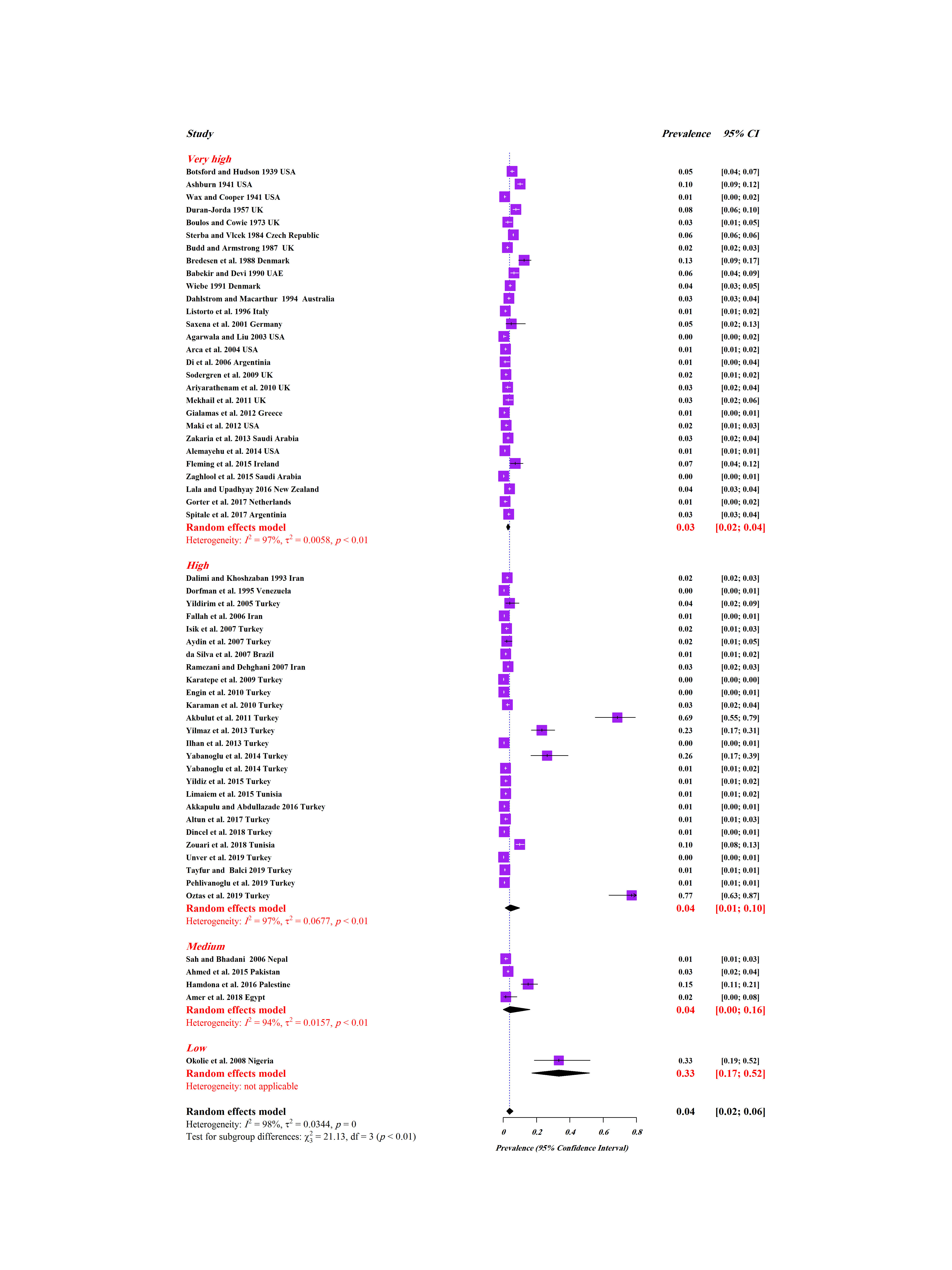

Supplement: S3 Fig — (JPG) [file pone.0232143.s003.jpg]
